# Photoacoustic Imaging for Image-guided Endovenous Laser Ablation Procedures

**DOI:** 10.1038/s41598-018-37588-2

**Published:** 2019-02-27

**Authors:** Yan Yan, Samuel John, Mahboobeh Ghalehnovi, Loay Kabbani, Nicole A. Kennedy, Mohammad Mehrmohammadi

**Affiliations:** 1 Department of Biomedical Engineering, Wayne State University, Detroit, MI 48202 USA; 2 Department of Electrical and Computer Engineering, Wayne State University, Detroit, MI 48202 USA; 3 Department of Vascular Surgery, Henry Ford Hospital, Detroit, MI 48202 USA

## Abstract

Accurate fiber tip tracking is a critical clinical problem during endovenous laser ablation (EVLA) of small perforating veins. Currently, ultrasound (US) imaging is the gold-standard modality for visualizing and for accurately placing the ablation fiber within the diseased vein. However, US imaging has limitations such as angular dependency and comet tail artifacts. In addition, EVLA is often performed without any real-time temperature monitoring, which could lead to an insufficient thermal dose or overheating the surrounding tissue. We propose a new technique that combines US and photoacoustic (PA) imaging for concurrent ablation fiber tip tracking and real-time temperature monitoring during EVLA procedures. Our intended implementation of PA imaging for fiber tracking requires minimal modification of existing systems, which makes this technology easy to adopt. Combining US and PA imaging modalities allows for simultaneous visualization of background anatomical structures as well as high contrast, artifact-free, and angle-independent localization of the ablation fiber tip. Preliminary data demonstrates that changes in the amplitude of the PA signal can be used to monitor the localized temperature at the tip of the ablation fiber, which will be invaluable during EVLA procedures. These improvements can enhance the physician’s accuracy in performing EVLA procedures and will have a significant impact on the treatment outcomes.

## Introduction

**Figure 1:**
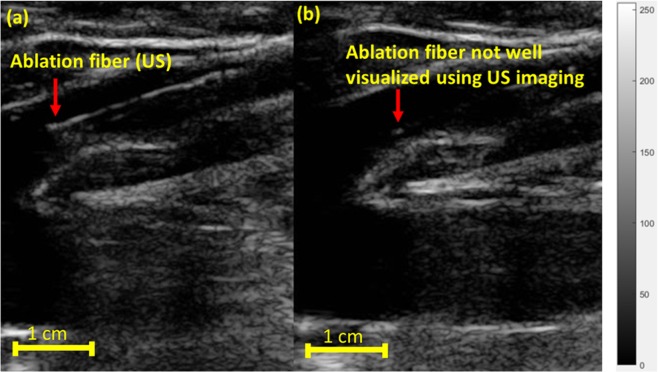
Sagittal US images visualizing the ablation fiber in a diseased vein during EVLA. (**a**) US imaging visualizes the ablation fiber inside the diseased vein. (**b**) Due to angular dependency and improper alignment of the US transducer with respect to the ablation fiber, it is not clearly visualized. Images were obtained from Henry Ford hospital (Detroit, Michigan).

It is predicted that nearly 20 percent of the adult population in the United States will develop varicose veins at some point in their lifetime^1^. The pathology of varicose veins is due to weakening of vein valves, which causes accumulation of blood and bulging within superficial veins. Currently, several invasive and minimally invasive^2^ treatment methods^3,4^ are used for the treatment of varicose veins. An example of an invasive treatment method is venous phlebectomy^5^, in which the vein is pulled out through a sequence of small incisions. Another invasive technique known as ligation, involves tying the veins through a small incision, thereby obstructing the accumulation of blood. There are other treatment methods such as sclerotherapy^6–8^, wherein the vein is injected with a solution or foam sclerosant which scars the endothelium and closes the vein. Minimally invasive treatment methods for venous insufficiency include radiofrequency ablation (RFA)^9^ and endovenous laser ablation EVLA^10,11^. In RFA, radiofrequency energy carried by an applicator is used to induce heat and seal the vein. In EVLA^12^, a fiber optic carrying a high power continuous-wave (CW) laser energy is inserted into the vein, and the localized heat produced at the fiber tip closes the diseased blood vessels^13^. For ELVA treatments, the 810 nm^11^, 940 nm, 980 nm^14^ and 1064 nm wavelengths are used for targeting blood absorption and the 1320 nm^15^ and 1470 nm^16^ wavelengths are used for heating the water-based vascular tissues. During EVLA procedures, real-time US imaging is used as gold-standard modality to help vascular surgeons visualize the position of the ablation fiber and the accurate placement of the ablation device within the diseased vein (Fig. 1a). The drawback with US imaging, however, is that it has some inherent artifact-related errors that limit its ability to track the fiber tip accurately. This is due to certain restrictions such as angular dependency^17^ and false readings of the fiber tip when it is angled out of the imaging plane^18^. Moreover, since the US image has a low signal to noise ratio (SNR) and a low contrast to noise ratio (CNR)^19^, it is somewhat difficult to identify the ablation fiber in the background image. Figure 1 demonstrates one of the challenges when using US imaging to track the ablation fiber inside the vein. Figure 1b indicates a scenario where improper alignment of the US probe with respect to the fiber leads to a severe angular dependency. Thus, the US image of the fiber and its tip are distorted and cannot be visualized.

Current EVLA systems lacks the ability to non-invasively determine the tissue temperature in real-time. Vascular surgeons apply a CW laser beam for a certain time interval, hoping to deliver enough energy locally within the diseased vein for proper ablation. In some EVLA cases, the high peak temperature inside the vein cause perforation of the vein wall and/or more perivenous damage, which may lead to increased pain and ecchymosis^14,20,21^. This perivenous damage becomes especially significant during the treatment of short superficial perforating veins. Due to the poor treatment of these superficial veins, varicose veins reoccur at a rate of 20 to 40 percent^22^. In particular, the unwanted thermal dose (heat) deposited into the ablated tissue has also been postulated to cause more endovenous heat induced thrombosis (EHIT)^23^. Currently, there is insufficient information about temperature rise both in the diseased vein and in the surrounding tissues. An uncontrolled heat dose can lead to the overheating of the surrounding tissues.

This research study aims to investigate the improvement of image-guidance in EVLA procedures for enhancing the quality of healthcare for patients with venous insufficiencies. In order to overcome these limitations of US-guided EVLA, there is a need for a non-invasive imaging modality for accurate visualization of the ablation fiber and real-time monitoring of the tissue temperature. The objective of this study is to investigate the utility of combined US and PA imaging to enhance EVLA guidance. PA imaging uses short, non-ionizing light pulses to excite the tissue of interest, which leads to the generation of acoustic waves through the tissue’s thermoelastic expansion^24–27^. Compared to US imaging which indicates the structural properties of the tissue^28^, PA imaging offers the functional and the molecular information of the tissue of interest^24–26^. The PA spherical acoustic waves that are emitted from the tissue can be detected by a US transducer, and they can be used to form a co-registered PA image. We propose to add a low power pulsed laser to the CW beam being carried by the ablation fiber and the use of PA imaging for tracking the fiber tip, since the PA signal is only generated at the interface between the fiber tip and the tissue (i.e. blood inside the diseased vein)^29^. Therefore, the PA image of the fiber tip will possess a very high CNR and more importantly, due to the omnidirectional radiation of PA waves, it will be less angular dependent. Also, the amplitude of the PA signal is proportional to the surrounding temperature^30^. Hence, we propose to use PA imaging for monitoring the temperature at the fiber tip in real-time. The combination of US and PA imaging would offer a significant benefit over traditional US imaging tools for guiding the ablation fiber and would require minimal modifications of the EVLA and US guidance devices.

## Results and Discussions

### Use of PA imaging for fiber tip tracking

**Figure 2:**
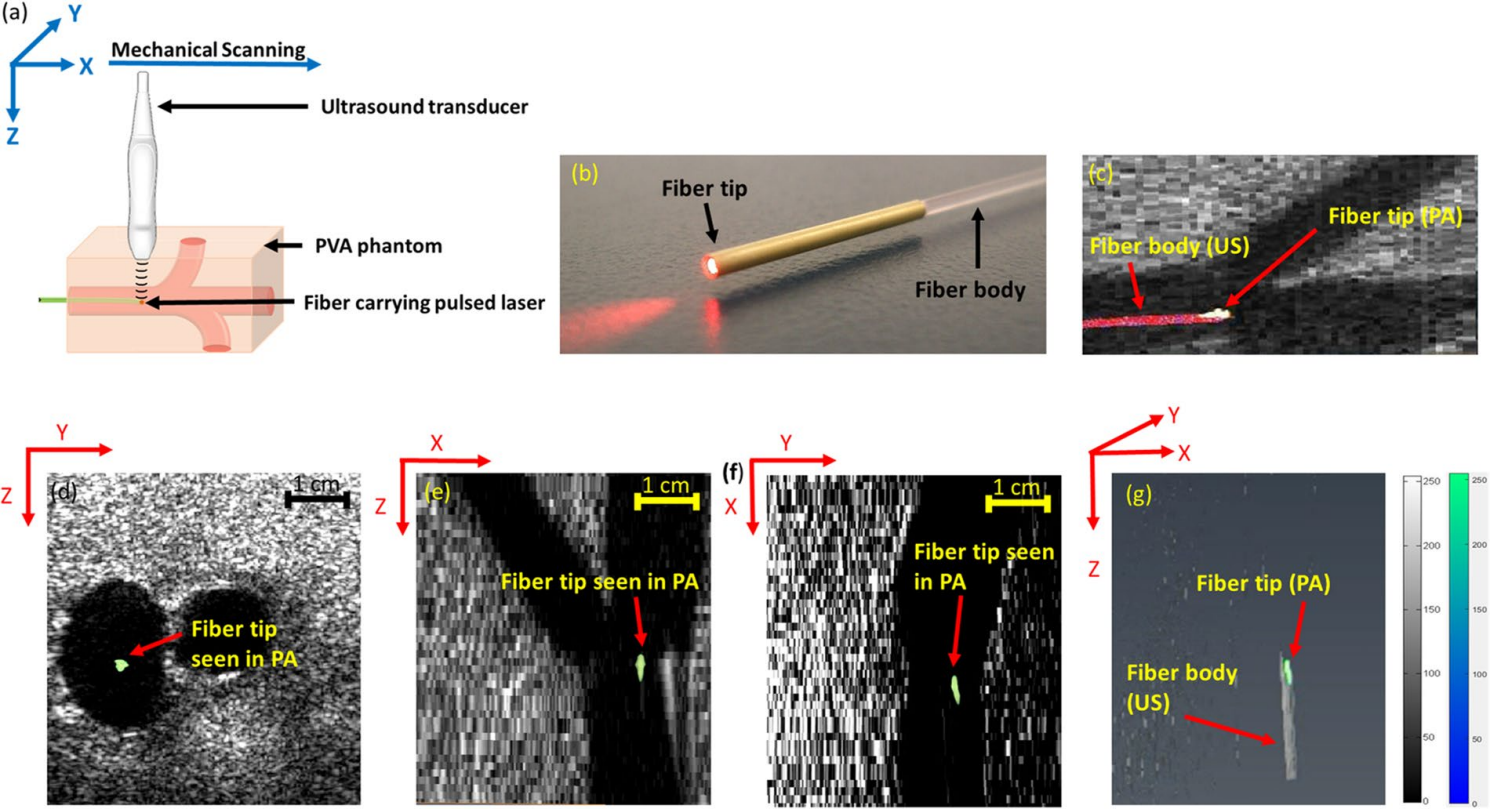
(**a**) Experimental setup for straight fiber tip tracking. (**b**) image showing the ablation fiber used, the ablation fiber has a gold covering at its tip. (**c**) Fiber tip seen using PA imaging, body of the fiber seen using US imaging. Appearance of the straight fiber in combined US and thresholded PA imaging in (**d**) transverse, (**e**) sagittal, and (**f**) coronal planes. (**g**) Volumetric image of the fiber in both US and thresholded PA images indicating that PA is only visualizing the fiber tip while US imaging shows the whole fiber body. Supplementary Movie 1 shows the volumetric US and PA of the phantom with straight fiber.

Figure 2 shows the US and the thresholded PA images of the fiber placed in the straight orientation (Fig. 2a) inside the phantom in transverse, sagittal, and the coronal planes (Fig. 2d–f), respectively. Straight orientation represents the scenario in which the ablation fiber is positioned parallel to the surface of the US transducer and thus no angular dependency issues are involved. The anatomical structure of the vascular-mimicking phantom is clearly visualized in the background B-mode US image and the PA image indicates the location of the fiber tip. While the body of the fiber is visualized using US imaging (Fig. 2c), PA signal only arises from the tip of the fiber and thus PA image visualizes only the fiber tip (Fig. 2d–g). A three-dimensional (3D) volumetric US/PA image of the phantom and straight fiber is constructed by 3D rendering of acquired 2D, equally distanced (1 mm) transverse slices (Fig. 2g). While US image provides an image of the fiber body, the PA image is only produced at the tip. In other words, while US images of the fiber body’s cross-section and the tip look similar and may cause false interpretation of the fiber tip location, PA imaging can help prevent such false readings. The tip of the fiber has a gold coated layer (Fig. 2b), which is a known, strong light absorber. This gold at the fiber tip affects the generation of the PA signal since the light exiting the fiber will be diffused inside the vessel and a portion of it will be absorbed by the gold layer. While gold acts as the strong light absorber, most of the PA signal is still generated from the immediate medium surrounding the gold layer (i.e. blood)^31^.

**Figure 3:**
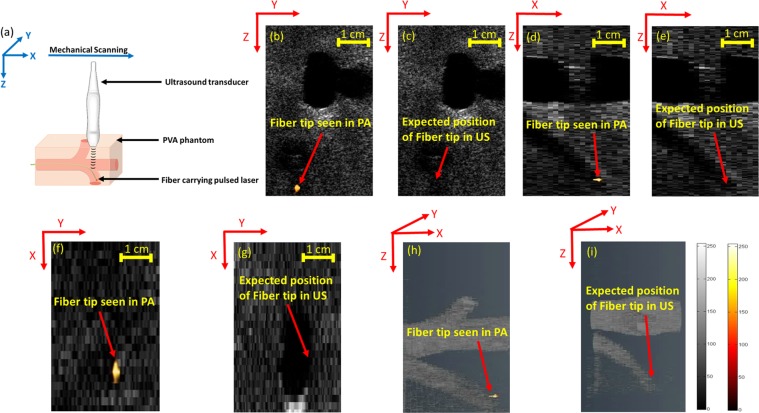
(**a**) Experimental setup for angled fiber tip tracking. US imaging of the angled fiber indicating the pulse-echo US limitations in tracking the angled fiber in (**c**,**e**,**g**,**i**). Thresholded PA imaging of the angled fiber inside vessel-mimicking phantom, superimposed over B-mode US image of the phantom in (**b**,**d**,**f**,**h**). Supplementary Movies 2 and 3 show the volumetric US and PA of the phantom with angled fiber.

Figure 3 compares the ability of US and PA to track the ablation fiber tip in an angled (45 degree) orientation (Fig. 3a) inside the phantom in the transverse, sagittal and the coronal planes (Fig. 3b–g). Due to the angular dependency, US imaging failed to locate the fiber (Fig. 3c,e,i). The echoes reflected from the angled fiber are directed outside the US imaging field of view (FOV) and are not acquired by the US transducer. In contrast, the accurate location of the fiber tip inside the angled vein is clearly visualized in the PA images (Fig. 3b,d,h) as the generated spherical PA waves, travel omnidirectionally and are eventually picked up by the US transducer. Figure 3h and i represent 3D volumetric US and combined US and PA images of the fiber placed inside an angled vein mimic and demonstrate the advantage of combining US and PA imaging to acquire both structural background and angular-independent image of the fiber tip.

**Figure 4:**
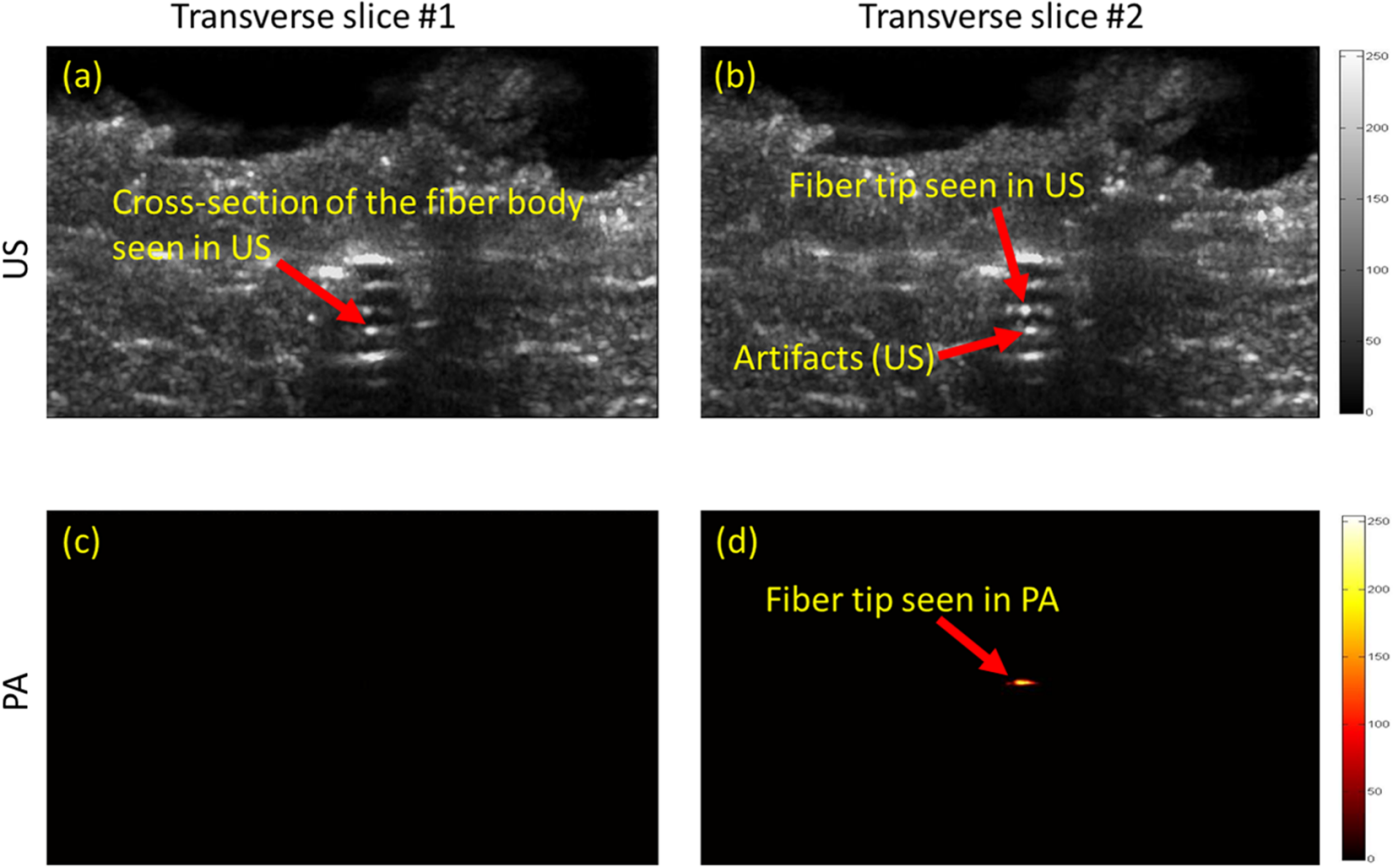
US (**a**,**b**) and PA (**c**,**d**) images of a fiber inside a porcine tissue in straight orientation. US imaging is unable to track the fiber inside the porcine tissue in (**a**,**b**). The accurate location of the fiber tip is clearly seen using thresholded PA imaging inside the porcine meat tissue in (**c**,**d**).

**Figure 5:**
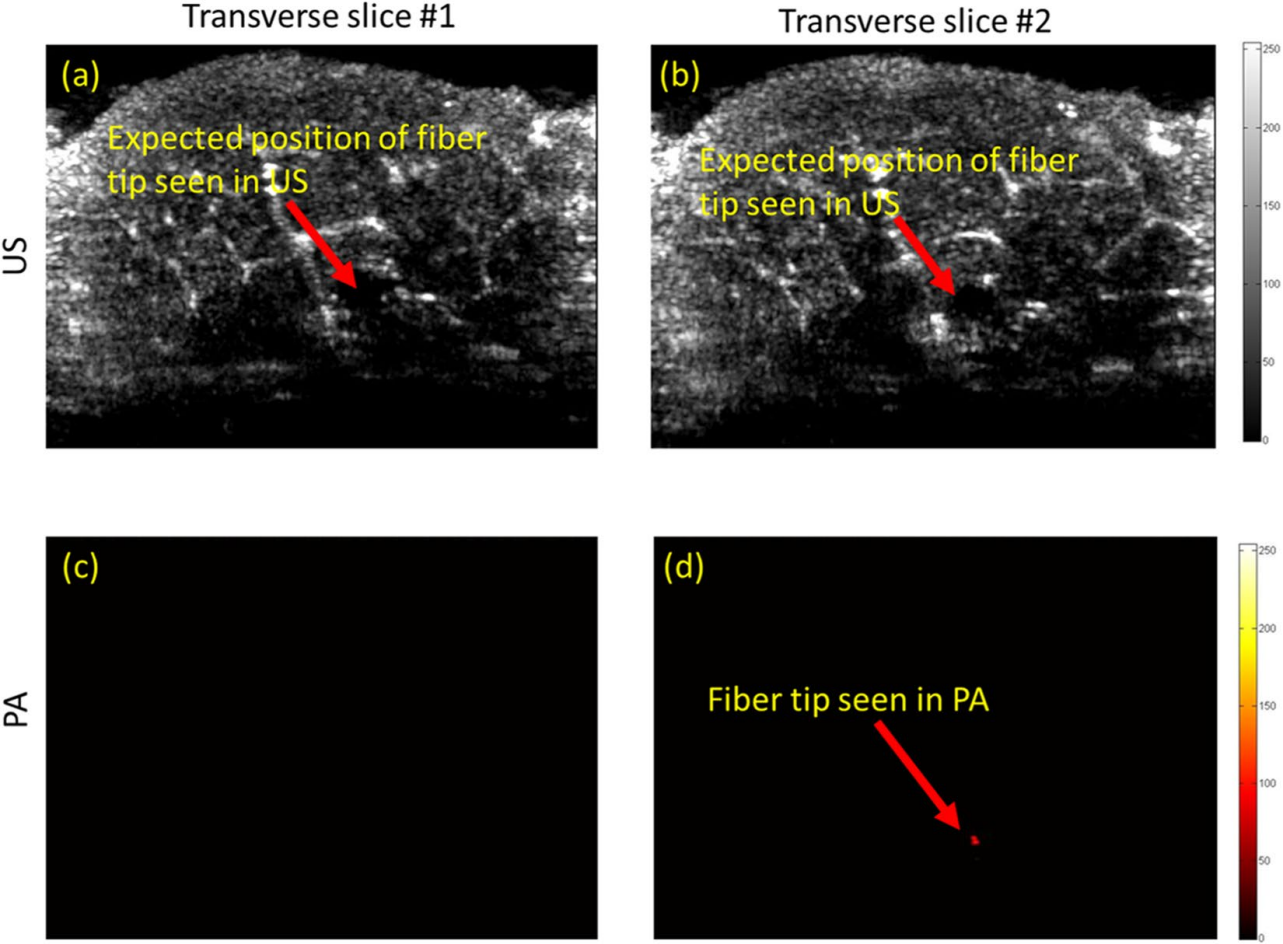
US (**a**,**b**) and PA (**c**,**d**) images of a fiber inside a porcine tissue at an angle of 30 degrees. Due to angular dependency issues, US imaging is unable to track the fiber inside the porcine tissue in (**a**,**b**). The accurate location of the fiber tip is clearly seen using thresholded PA imaging inside the porcine tissue in (**c**,**d**).

In order to further examine the performance of PA in providing high contrast and angular independent images of the fiber tip, a set of experiments were performed in which a fiber was placed inside a US transparent tube of diameter of 4 mm, and then was embedded inside a porcine tissue serving as the background. Figure 4 compares the transverse US and the thresholded PA images of the fiber placed in the straight orientation inside the porcine tissue. Twenty transverse slices of the porcine tissue were imaged during the experiment. Figure 4 shows two selected US and PA slices, one was chosen at 1 mm behind the tip of the fiber (Fig. 4a,c), and the second one was chosen at the fiber tip (Fig. 4b,d). The results show that the similarity of US images of the fiber body cross-section and its tip (Fig. 4a,b), makes it difficult to identify the fiber tip location. However, as anticipated, PA signal is only generated at the tip (Fig. 4d) and thus no PA signal was detected from the body of the fiber (Fig. 4c). In other words, PA imaging does not suffer from the possible false positioning of the fiber tip due to its similar appearance to the fiber body, as it can occur in US imaging. The anatomical structure of the tissue was clearly visualized using US imaging (Fig. 4a,b), while PA imaging revealed a background-free image of the fiber tip. The SNR (Eq. 4) and the CNR (Eq. 5) of US and the thresholded PA images of the fiber tip were calculated and compared. The noise floor of PA image was estimated using histogram analysis and was set to 18.75 percent of the maximum PA signal produced the tip of the fiber. PA signal values below the threshold were masked to zero (Supplementary Document Fig. 4). The SNR values of the US and thresholded PA images of the tip were calculated as 4.047 dB and 7.978 dB respectively. Since in US imaging, both background tissue and the fiber are imaged through identical pulse-echo mechanism, somewhat similar signal intensity reflected from the fiber and the vessel-mimicking tube, causes a low CNR of −6.626 dB. In contrast, the high contrast PA image of the fiber tip led to a significantly higher CNR of 8.302 dB (Supplementary Document, Section 3). Figure 5 compares the transverse US and the thresholded PA images of the fiber placed in an angled orientation (30 degrees) inside the porcine tissue. While the fiber tip was not seen in US images due to the angular dependency (Fig. 5a,b), PA was able to locate it with a high contrast (Fig. 5d).

**Figure 6:**
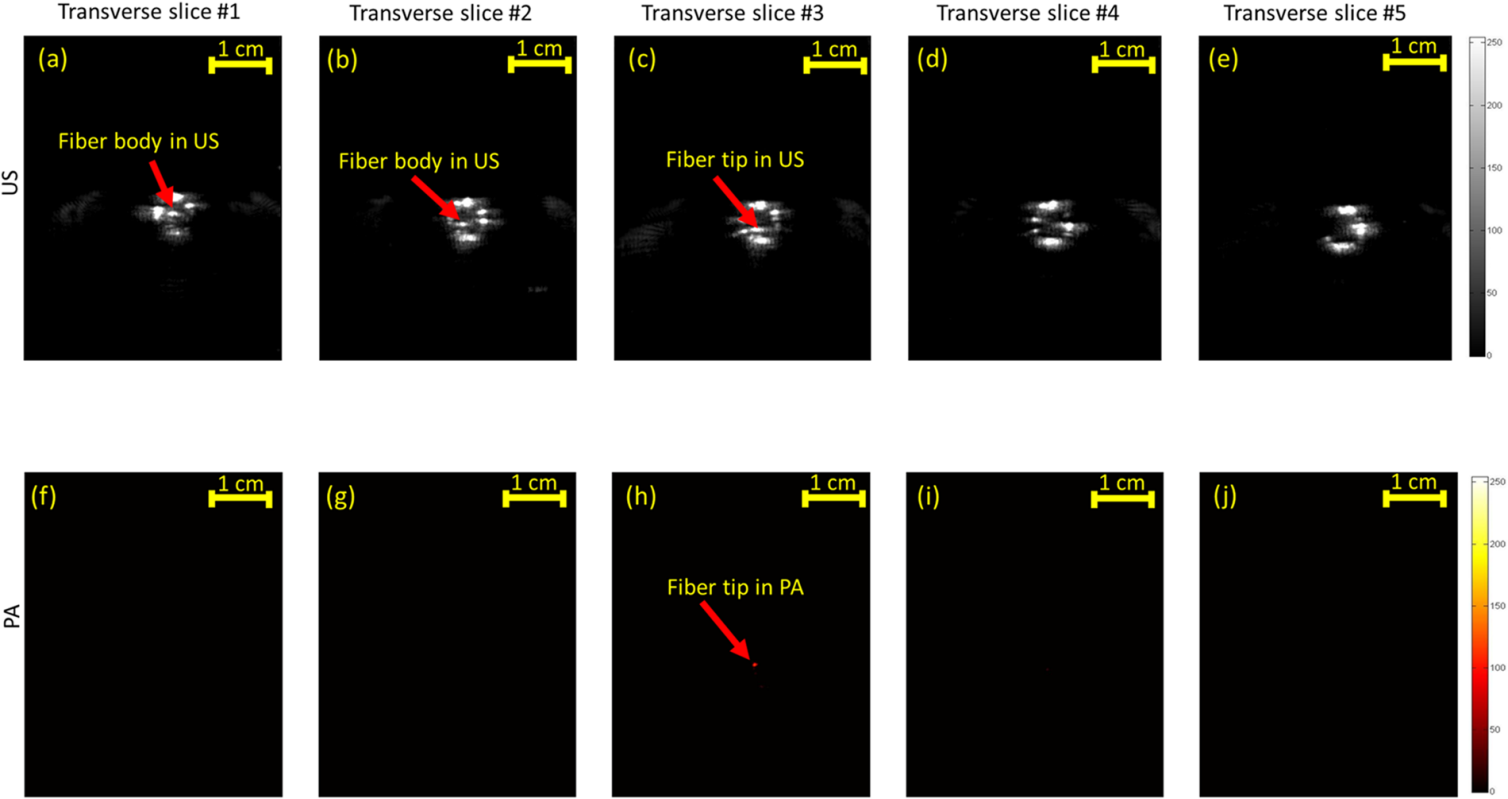
US (**a**–**e**) and PA (**f**–**j**) mechanically scanned transverse images of a fiber tip placed inside a blood-filled tube at behind (panels **a–b** and **f–g**) of the tip, at the fiber tip (panels **c,h**) and after passing the fiber (panels **d–e**, and **i–j**). US imaging is unable to distinguish between the fiber tip and the cross section of the fiber in (**a**–**c**). The accurate location of the fiber tip is clearly seen using thresholded PA imaging in (**h**).

The ability of US and PA imaging to distinguish between the fiber tip and the cross-section of the fiber was further evaluated in a set of experiments in which the fiber was placed inside a US transparent tube filled with heparinized sheep blood (Cedarlane, Ontario, Canada - refer to Supplementary Document). Similar to the previous experiment, twenty transverse US and PA images of the tube cross-section were mechanically scanned with a scan step size of 2 mm. Since the fiber was placed inside the blood medium, the pulsed energy laser was lowered down to 100 *µJ* and yet strong PA signals were acquired. For each modality, five transverse planes (two behind the fiber tip, one at the tip and two after passing the fiber tip) were chosen and are shown in Fig. 6. The similar appearance of fiber body and fiber tip in US images are depicted in Fig. 6a–c. However, the tip was only visualized in one of the transverse PA images in which the US probe was aligned above the fiber tip. It must be noted that the transverse imaging of the tubes and the relatively large mechanical scan steps of 2 mm prevent the strong appearance of the fiber tip in the images acquired before and after the tip. With smaller scanning steps, one could anticipate receiving a weaker PA signal in imaging slices adjacent to the peak due to their omnidirectionality. Using relatively low energy laser pulses, PA image of the fiber tip appeared with the SNR of 6.598 and CNR of 6.130 dB. Such high SNR and CNR values indicate the feasibility of guiding EVLA fibers in a blood medium with an addition of a low-cost 532 nm pulsed laser.

### Use of PA imaging for thermometry

**Figure 7:**
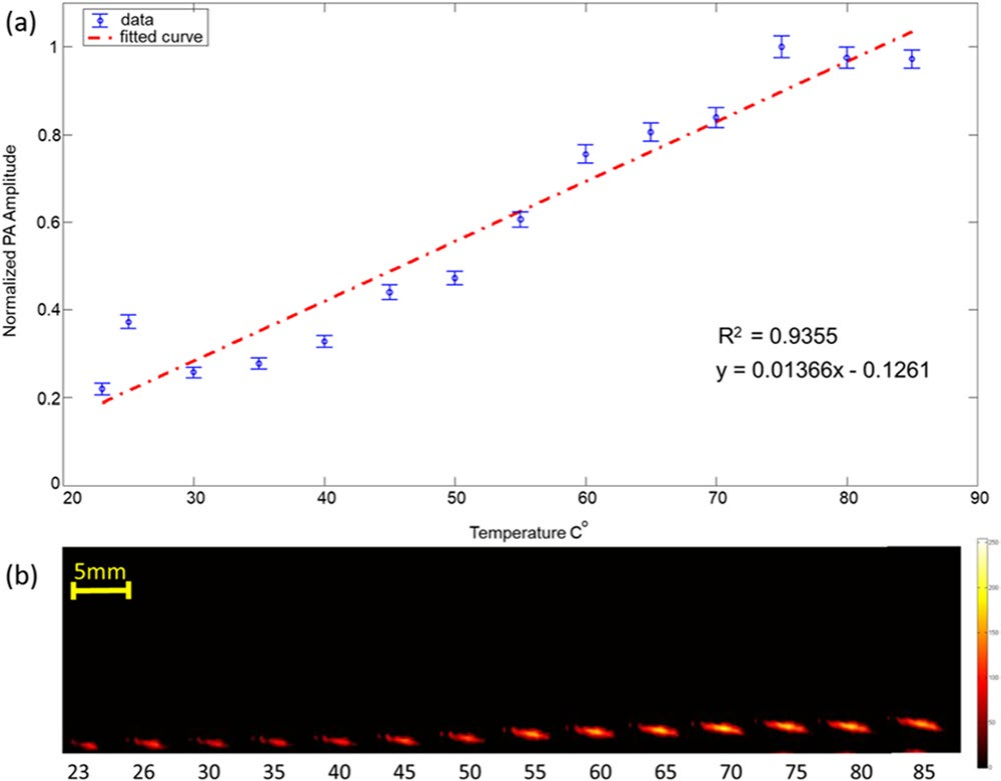
(**a**) PA imaging to monitor the changes in amplitude of the PA signal with increase in the surrounding temperature. (**a**) Plot indicating normalized amplitude of the PA signal obtained versus surrounding temperature, blue dots indicate measurements and the red dashed line represents the linear fit. (**b**) PA images of the fiber tip at different surrounding temperature. The increase in PA amplitude as well as the effect of speed of sound variation can be clearly seen in (**b**).

The potential ability of PA for real-time monitoring of the temperature at the fiber tip was evaluated in a set of *ex vivo* experiments in which the temperature of the medium surrounding the fiber tip (water) was altered during PA image acquisition. An ablation fiber was placed inside a tubing system through which temperature-controlled water was flowing. The water temperature was changed between 23 to 85 °C . Acquired PA signals at different temperatures were normalized to their maximum values and were plotted (Fig. 7a). A steady increment in the amplitude of the PA signal was observed (*R*^2^ = 0.935) when the surrounding temperature was increased. The increment in the amplitude of the PA signal originated from the increase in the Grüneisen parameter of the water at higher temperatures. In addition, the speed of sound increase in water leads to dislocation of the PA image of the fiber tip (Fig. 7b). Compared to PA thermometry procedures utilizing external illumination^32–39^, the proposed method benefits from a significant advantage in which the laser fluence (energy at the output of the fiber) is constant. The initial PA pressure wave (i.e. PA signal) is defined by^40^

1 $$P={\rm{\Gamma }}{\mu }_{a}F$$

where Γ corresponds to the Grüneisen parameter, *µ*_*a*_ is the absorption coefficient, and laser fluence is denoted by F. Therefore, the amplitude of the PA signal is proportional to the product of the fluence, the Grüneisen coefficient, and the absorption coefficient of the tissue. Quantitative PA thermometry requires an accurate estimation of the laser fluence at the desired target which is often a challenging task due to the complexity of accurate estimation of the light diffusion inside heterogeneous tissues. In our method, as the PA signal is generated from the interface of the fiber tip and the surrounding medium (blood), the fluence remains constant. Therefore, in contrast to external illumination PA thermometry methods, our technique has fewer difficulties providing accurate quantitative measurements of the temperature of the tissue. It is also known that the increase in temperature above 50 °C causes the denaturation and coagulation of blood, eventually producing carbon particles^41,42^. All these phenomena cause the changes in the optical absorption properties of blood. However, by measuring the optical properties of blood at higher temperatures, it is feasible to compensate for these variations and thus perform quantitative thermometry. Our thermometry system can be calibrated once, i.e. measure the relation between temperature of blood and PA signal at a constant pulsed energy; and this baseline information can be used to provide quantitative temperature measurements in veins located in different tissues and at different depths. Future studies are required to determine the relation between PA signal and the temperature of the blood within the range of the temperatures occurring during the ablation. These calibration studies will study the effects of the blood denaturation on the measured PA amplitude signal with an eye towards determining the absolute temperature at the fiber tip in relation to commercial thermometers.

### An integrated EVLA/US PA imaging system

**Figure 8:**
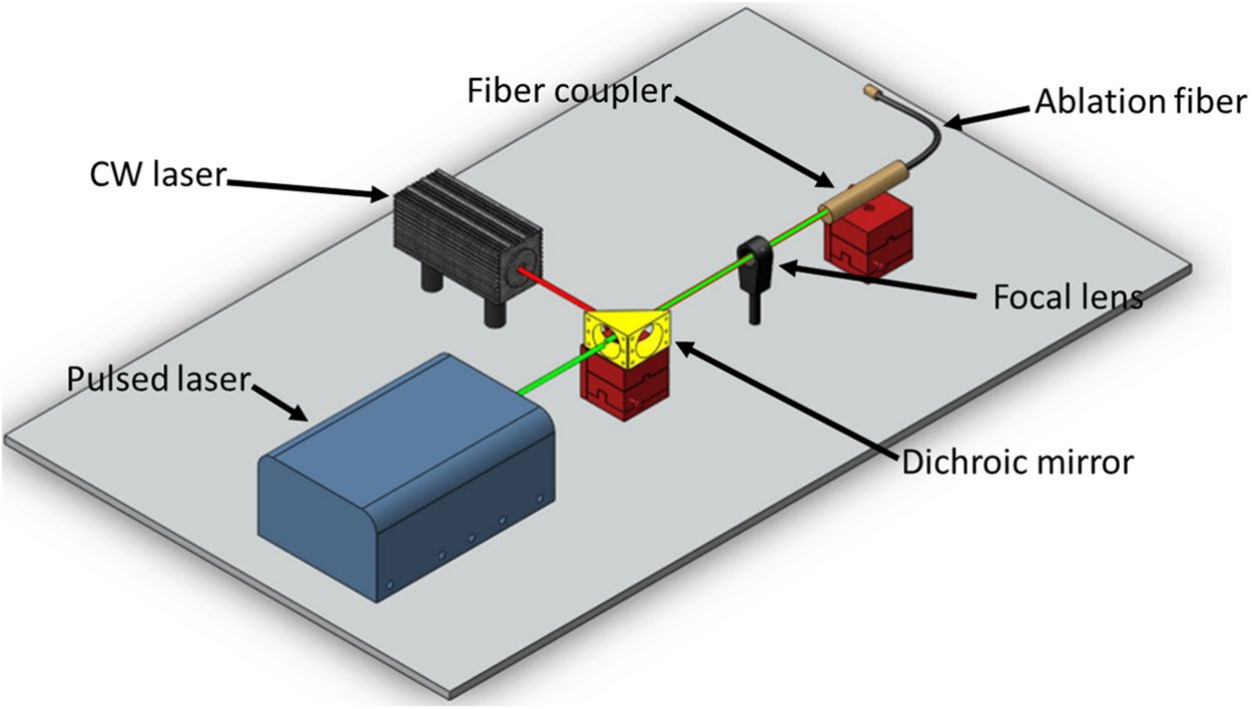
Block diagram of the combined PA and EVLA system. The ablation fiber carries the combined beam from the pulse laser and the CW ablation laser through a dichroic mirror and provides simultaneous ablation fiber tip tracking and real time temperature monitoring inside the vein. Supplementary Movie 4 shows the combined CW laser beam (λ = 808 nm) and pulsed laser beam (*λ* = 532 nm) using proposed optics.

The proposed PA imaging system can be easily integrated with the currently used US-guided EVLA systems because: (a) PA signals can be acquired by the same US transducer as used in US-guidance; (b) a pulsed laser excitation required for PA imaging can be easily coupled into the fiber carrying CW ablation beam. Since the required wavelengths for EVLA (*λ* = 1470 nm) and PA (532 nm) imaging are spectrally apart from each other, low-cost optical components such as dichroic mirrors can be utilized to combine CW and the pulsed laser beams into a single fiber (Fig. 8). Using an off-the-shelf shortpass dichroic mirror (DMSP 650, Thorlabs, Newton, NJ) with cut-off bandwidth of 650 nm, we were able to combine a 532 nm pulsed beam and a CW 808 nm (for the sake of visibility of the beam) into an ablation fiber (Supplementary Movie 3). Similar dichroic optics (with larger damage threshold tolerance) can be used to combine standard 1470 nm ablation lasers and a 532 nm pulsed laser for practical applications. Since the power of the pulsed laser is orders of magnitude lower than the ablation laser, an addition of a pulsed laser for PA-guided EVLA does not need additional safety concerns.

The addition of PA to US imaging for the guidance of EVLA procedures is also beneficial because US and PA images are naturally co-registered. There is no need for any complicated image processing for superimposing PA on US images. Compared to the current US-guided EVLA procedures, visualization of the fiber tip in PA imaging is angle-independent and a significantly improved CNR makes the visualization easier for the surgeons. Unlike US imaging, PA can easily differentiate between the fiber tip and the cross section of the fiber body which can reduce the false readings of the tip during the treatment procedures. Lastly, PA offers a unique ability to monitor the temperature at the tip of the fiber which can serve as crucial information to determine the thermal dose deposition and monitor the tissue ablation. The current US-guided EVLA systems can be easily modified by combining pulsed and CW laser beams into a single ablation fiber to perform simultaneous ablation, accurate fiber tracking and real-time temperature monitoring. These modifications can be made at low-cost and will be easy-to-adopt for clinicians since they do not significantly change the imaging and ablation procedure.

## Methods

### Principles of PA imaging

In PA imaging, a short laser pulses (in order of nanoseconds) are used to irradiate the tissue^24,43^. The light energy absorbed by the tissue is converted to heat and causes thermoelastic expansion within the tissue^40^. Since the energy deposition in tissue occurs in a short period (less than few tens of nanoseconds), the rapid expansion of the tissue generates broadband, spherical acoustic waves traveling omnidirectionally from the source. These acoustic waves can be detected by clinical US transducers and provide information about the optical properties of the tissue^24–26^. When an ablation fiber is inserted into the tissue (for example into veins), a pulsed excitation generates a strong signal at the tip of the fiber where the light enters the tissue and has its strongest intensity (fluence). Since a US transducer can be used to acquire both US and PA signals and both modalities are using similar image formation processes, combined US and PA imaging can provide co-registered US images of tissue background overlaid by PA image of the ablation fiber tip. It is also known that the amplitude of the PA signal varies when the surrounding temperature changes. The Grüneisen coefficient (Eq. 1) can also be written as:

2 $${\rm{\Gamma }}(T)=\frac{\beta {c}^{2}}{{C}_{p}}({\rm{T}})$$

where *β* is the thermal coefficient of volume expansion, *c* corresponds to the speed of sound and *C*_*p*_ is the heat capacity at constant pressure.

3 $${\rm{\Delta }}T=a\,\frac{{\rm{\Delta }}P}{P}$$

where $\frac{{\rm{\Delta }}P}{P}$ refers to the relative amplitude of the PA signal, *a* refers to the temperature dependent constant and ∆*T* refers to the change in temperature of the surrounding medium.

When a fiber tip is placed inside the vein, the change in the amplitude of the PA signal determines the temperature at the fiber tip. This is directly proportional to the thermal dose deposited within the venous tissue.

### Combined US and PA imaging for fiber tip tracking

We proposed using combined US and PA imaging for accurately guiding the ablation fiber into the perforating veins. This would be achieved by combining the pulsed laser beam (PA imaging) and the CW laser beam (ablation) through a dichroic mirror into an ablation fiber. By overlaying the PA signal onto the US anatomical data, one can clearly identify the location of the fiber tip. The capability of the PA - US imaging system in tracking the fiber tip was evaluated by: (a) tracking the accurate location of fiber tip in straight and angled orientations using tissue-mimicking phantoms and porcine tissues, (b) and by distinguishing between the fiber tip and the cross section in vein analogue phantoms filled with heparinized fresh sheep blood.

### Ability of PA imaging in tracking the accurate location of fiber tip in straight and angled orientations using tissue-mimicking PVA phantoms and porcine tissues

The experimental setup consisted of a portable tunable laser system (PhocusCore, Optotek, California, USA) of wavelength (*λ* = 532 nm), repetition rate of 10 Hz coupled to an ablation fiber (NeverTouch, Angiodynamics, New York, USA) of diameter 1000 *µm*. A high frequency linear array US transducer L11-4V (128 element, Verasonics, Washington, USA) is used to receive the US and PA images with the programmable digital US research platform (Vantage 128, Verasonics, Washington, USA). The acquisition software used for acquiring US and PA images is the Vantage software (Verasonics, Kirkland, Washington, USA) During our experiments, one wide-beam compounded US image was acquired as the background between each PA acquisition. A mechanical XY scanning platform (MICOS Mini-80 XY stage, PI MICOS gmbh, Massachusetts, USA) was used for moving the US transducer across the phantom and porcine tissues in both pulse echo US and PA modes.

The polyvinyl alcohol (PVA) vessel-mimicking phantom consisted of two straight and angled vessel-mimicking channels filled with water to simulate the perforating veins. The fiber carrying the pulsed laser of wavelength (*λ* = 532 nm) and pulse energy of 100 *µJ*, was placed inside the phantom in two different orientations: (a) straight, meaning that the fiber was placed perpendicular to the incident US beam and (b) angled meaning that the fiber was placed at angle of 45 degrees incident to the US beam. Equidistant (1 mm) US and PA images were obtained by mechanically scanning the US transducer over the surface of the phantom. The acquired multi-slice US and PA images are processed using a commercial 3-D rendering software to construct the volumetric images for analyzing the ability of PA in tracking the fiber tip in straight and angled orientations.

For porcine tissue experiments, the fiber coupled to the pulsed laser of wavelength (*λ* = 532 nm) and pulse energy of 200 *µJ*, connected to an ablation fiber was inserted into a tube filled with water, placed inside the porcine tissue in straight and angled orientation (30 degrees) (Figs 2a and 3a). US coupling gel was applied onto the surface of the tissue to facilitate easy acquisition of US and PA signals through the US transducer. The US transducer was mechanically scanned with a scan step size of 1 mm over the surface of the phantom to acquire US and PA images at different cross-sections.

### Ability of PA imaging in distinguishing between the fiber tip and the cross section of the fiber in vessel-mimicking phantoms filled with heparinized fresh sheep blood

The inability of US imaging to distinguish between the fiber tip and the cross section of the fiber was evaluated by placing the fiber coupled with pulsed laser of wavelength (*λ* = 532 nm) and pulse energy of 100 *µJ* in an US transparent tube filled with heparinized fresh sheep blood (Cedarlane, Ontario, Canada). US and PA images at different cross sections of the tube were obtained by mechanically scanning the US transducer with a scan step size of 2 mm along the surface of the tube placed in a water medium (refer to Supplementary Fig. 1).

### Calculation of SNR and CNR for US and thresholded PA images for porcine tissue studies and vessel-mimicking phantoms filled with heparinized fresh sheep blood

The SNR and CNR of US and the thresholded PA images were calculated and compared for porcine tissue experiment (Fig. 4b,d) and vessel-mimicking phantoms filled with heparinized fresh sheep blood (Fig. 6c,h) (refer to Supplementary Document, Section 1). SNR was defined through:

4 $$SNR=10\,{lo}{{g}}_{10}\frac{{{\rm{\mu }}}_{signal}}{{{\sigma }}_{background}}$$

where *µ*_*signal*_ refers to the mean signal generated at the tip of the fiber and *σ*_*background*_ refers to the standard deviation of the background (refer to Supplementary Document, Section 3). CNR was defined through

5 $$CNR=10\,{lo}{{g}}_{{\rm{10}}}\frac{|{S}_{{A}}-{S}_{{B}}|}{{\sigma }_{background}}$$

where *S*_*A*_ refers to the mean of the desired signal (fiber tip), *S*_*B*_ refers to the mean of the background (background tissue) and *σ*_*background*_ represents the background noise, measured through the standard deviation of the background signal (US transparent tube).

### Real-time PA Imaging Thermometry

The ability of the PA imaging system for monitoring the real-time temperature inside the tissue was evaluated by placing the fiber coupled with pulsed laser inside an US transparent tube (refer to Supplementary Fig. 2). A k-type thermocouple with a temperature range of (−40 to 250 °C) was placed in close proximity to the fiber tip inside the tube, and the temperature of the water inside the tube was increased from 23 to 85 °C. The two ends of the tube were firmly attached to the walls of the imaging tank filled with water, and the temperature rise inside the tube was acquired by averaging the PA signals generated at the tip of the fiber. This was compared to temperature measurements using a standard thermometer.

## Supplementary information

Supplementary Document

3D volumetric image of the fiber tip in straight orientation

3D volumetric image of the fiber tip in angled orientations using combined US and PA imaging

3D volumetric image of the fiber tip in angled orientations using US imaging

Ablation fiber carrying the combined continuous wave (CW) and pulsed beams

## Data Availability

The data that supports the findings of this study are available from the corresponding author on request.
