# Supplementary material for: Photoacoustic Imaging for Image-guided Endovenous Laser Ablation Procedures
Source: Sci Rep. 2019 Feb 27;9:2933. doi: 10.1038/s41598-018-37588-2 (PMC6393544; doi:10.1038/s41598-018-37588-2)
Supplement: Supplementary file 1 — Supplementary Document [file 41598_2018_37588_MOESM1_ESM.pdf]

Supplementary Material

Photoacoustic Imaging for Image-guided Endovenous Laser Ablation Procedures

Yan Yan[1, +], Samuel John[1, +], Mahboobeh Ghalehnovi[1], Loay Kabbani[3], Nicole A. Kennedy[3], Mohammad Mehrmohammadi[1,2,*]

[1]Department of Biomedical Engineering, Wayne State University, Detroit, MI, USA, 48202
[2]Department of Electrical and Computer Engineering, Wayne State University, Detroit, MI, USA, 48202
[3]Department of Vascular Surgery, Henry Ford Hospital, Detroit, MI, USA, 48202
[*]mehr@wayne.edu
[+]These authors contributed equally to this work

The supplementary document consists of the following:

1. Tissue-mimicking phantom experimental setup to distinguish between the fiber tip and the cross section of the fiber body using ultrasound (US) and photoacoustic (PA) imaging.
2. Experimental setup to validate the potential application of PA imaging for real-time temperature monitoring.
3. Calculation of signal-to-noise ratio (SNR) and contrast-to-noise ratio (CNR) for US and thresholded PA images of fiber tip placed inside a blood-filled vessel-mimic and inside a porcine tissue background.
4. Supplementary movies demonstrating:
    a) 3D volumetric US and PA images of the fiber tip placed inside a tissue-mimicking phantom filled with water in the straight and angled orientation.
    b) Combined beam (continuous wave (CW) laser at 808 nm + pulsed laser at 532 nm) carried by the ablation fiber.

1. Experimental setup to distinguish between the fiber tip and the cross section of fiber body using US and PA imaging.

The ability of PA imaging to differentiate between the fiber tip and the cross section of the fiber body is evaluated by inserting an EVLA fiber carrying the pulsed laser of wavelength λ =532 nm (pulse energy of 100 μJ) into an US transparent tube of diameter 4 mm filled with heparinized fresh sheep blood (Cedarlane, Ontario, Canada). The tube was placed inside a water tank as shown in **Supplementary Figure 1**. Transverse US and the PA images were obtained by mechanically scanning the US transducer over the surface of the tube. The scanning resolution (distances between imaging planes) was set to 2 mm **(Supplementary Fig. 1)**. Compared to US imaging, PA provides background-free images of the fiber tip **(Fig. 6f-j in the manuscript)**. US imaging is unable to distinguish between the fiber tip and cross section of the fiber body because they have similar appearance in US images **(Fig. 6a-e in the manuscript)**. Also, as the fiber is located within the heparinized fresh sheep blood, it generates the stronger PA signal at the peak absorption of blood (around 532nm) compared to the experiments in which the fiber was placed inside the water.

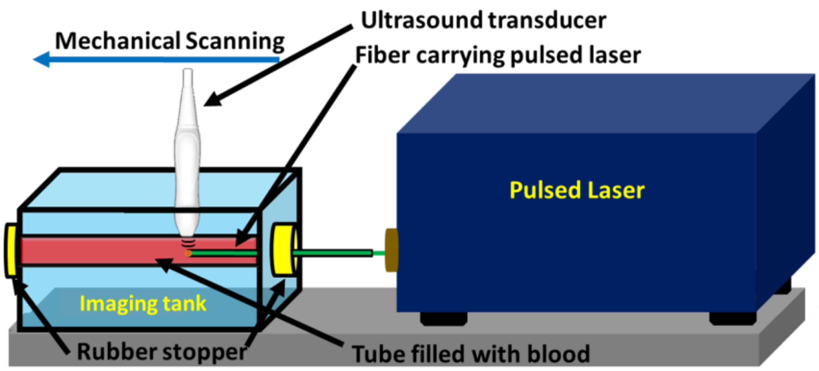

Supplementary Figure 1: Schematic representation of experimental setup to compare the US and PA imaging to distinguish between the fiber tip and the cross section of the fiber body.

2. Experimental setup to validate the potential application of PA imaging for real-time temperature monitoring.

The ability of the PA imaging to monitor the temperature at the tip of the fiber in real time was evaluated by placing a fiber coupled carrying pulsed laser (λ = 532 nm) inside a US transparent tube. The two ends of the tube are firmly attached to the walls of a container filled with water maintained at room temperature. A k-type thermocouple with a temperature range of -40 to 250°C was placed in close proximity to serve as the gold-standard thermometer for validation of PA measurements. Temperature controlled heated water was circulated through the tube and temperature of the water was controlled using a thermostat. The temperature of the water was increased from 23 to 85°C in a separate reservoir (water heating tank). PA images of the fiber tip were acquired at different temperatures of water running inside the tube by using a linear US transducer (L11-4V) imaging the transverse cross section of fiber and is aligned at its tip **(Supplementary Fig. 2)**. A clear increment in the amplitude of the PA signal was observed with rise in the ambient temperature **(Fig. 7 in the manuscript)**. Hence, PA imaging can potentially be used for real-time temperature monitoring inside the diseased vein.

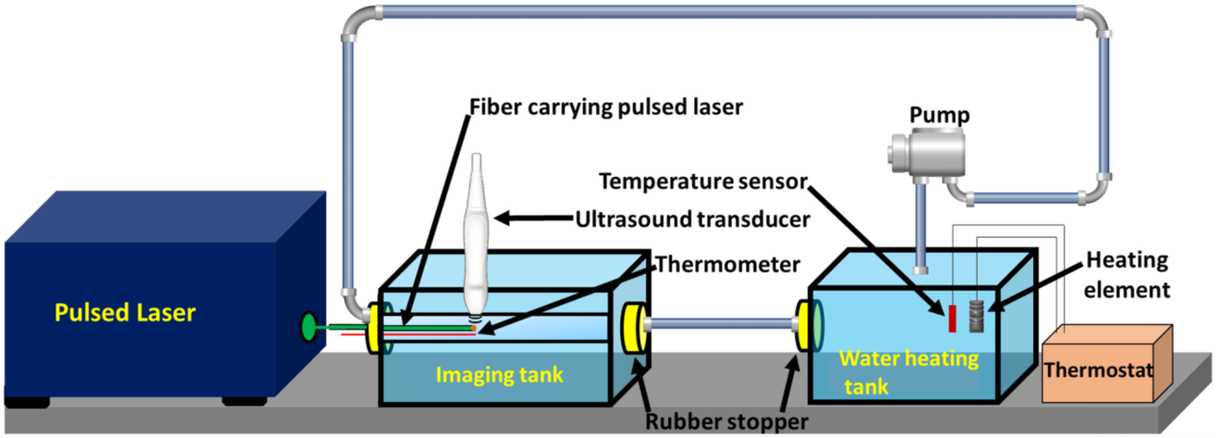

Supplementary Figure 2: Schematic representation of real-time temperature monitoring using PA imaging to study the changes in amplitude of the PA signal with surrounding temperature

3. Calculation of signal-to-noise ratio (SNR) and contrast-to-noise ratio (CNR) for US and thresholded PA images of fiber tip placed inside a vessel mimicking phantom filled with blood and inside a porcine tissue background.

The SNR and the CNR values for both US and PA images of the fiber tip placed inside a vessel-mimicking US transparent tube, filled with blood and embedded into a porcine tissue background **(Figures 4 and 6 in the Manuscript)**. The signal emerging from the fiber tip was considered as the object and the signal generated within the vessel-mimicking tube was considered as background. The SNR of the US and thresholded PA images were calculated as SNR= $\frac{Mean(object)}{std(background)}$ and CNR= $\frac{Mean(object)-Mean(background)}{std(background)}$, where *Mean(object)* and *Mean(background)* represent the mean (average) pixel values located inside the object and *std(background)* represents the standard deviation of the signal from the background region (in this case, the vessel-mimicking tube filled with blood). **Supplementary Figures 3 (a) and (b)** shows the selected regions of interest (ROIs) for background and fiber tip in US and the PA images.

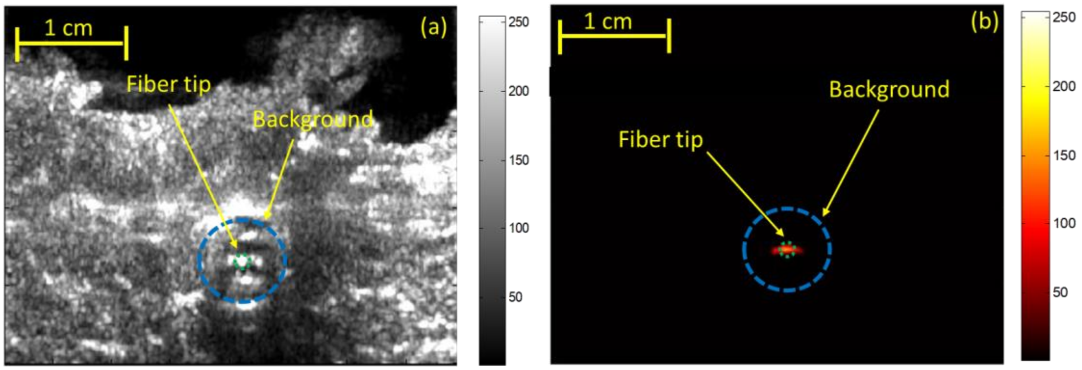

Supplementary Figure 3: Selection of the ROI for the background and the fiber tip for the (a) US and (b) thresholded PA image for porcine tissue. The blue dashed circle indicates the region selected as the background (in this case, inside the vessel-mimicking tube), and the green dotted circle indicates the fiber tip (object).

The noise floor of the PA image is calculated as 18.75% of the maximum PA image, based on the histogram analysis of the image (local minimum). PA signal intensities below the threshold were masked to zero (**Supplementary Figures 4 (c)).** For the case of straight fiber, the SNR for the US image is 4.047 dB and for thresholded PA image is 7.978 dB. The CNR of the US image was measured as -6.626 dB (very low due to presence of strong US signals inside the vessel mimic), while it is significantly improved in thresholded PA image (8.302 dB). **Supplementary Figure 4 (a)** shows the non-thresholded PA image, **(b)** shows the thresholded PA image and **(c)** histogram with calculation of local minimum value for calculating the noise floor.

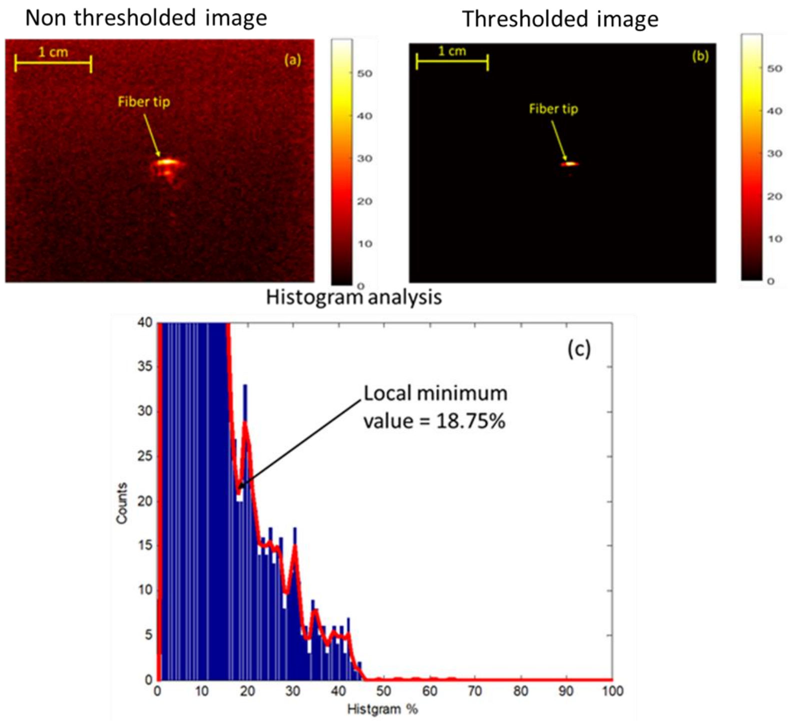

Supplementary Figure 4: Calculation of the noise floor of the PA image (a) non thresholded PA image, (b) thresholded PA image and (c) histogram with calculation of local minimum value at 18.75% maximum intensity

Same process was used to measure CNR and SNR of the fiber placed inside the vessel mimicking phantom filled with blood (**Figure 6c and 6h in the manuscript**). The SNR for the US image is 5.477 dB and the thresholded PA image is 6.598 dB. The CNR for US image is 0.888 dB and for thresholded PA image is 6.130 dB (**Supplementary Figure 5).**

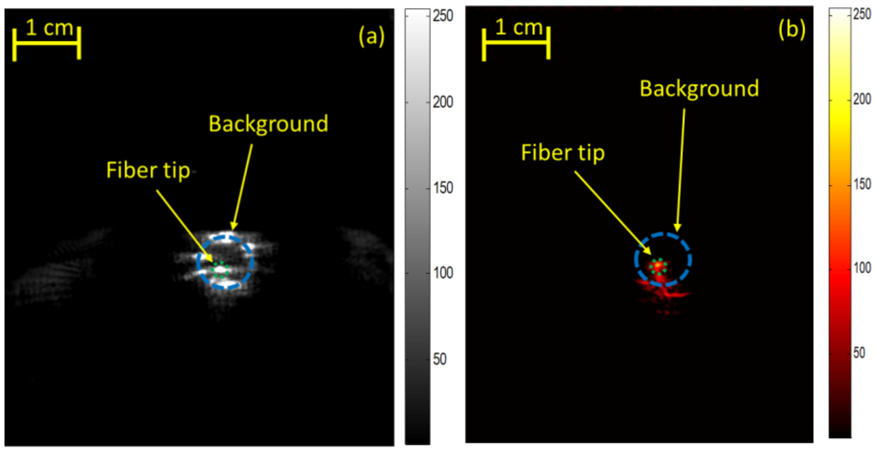

Supplementary Figure 5: Selection of the ROI for the background and the fiber tip for the (a) US and (b) thresholded PA image for vessel-mimicking phantoms filled with heparinized fresh sheep blood. The blue dashed circle indicates background (tube filled with blood) and the green dotted circle indicates the location of the fiber tip.

4. Supplementary movies:

A. 3D volumetric US and PA images of the fiber placed inside a tissue-mimicking phantom filled with water in the straight and angled orientations.

PA imaging provides artifact-free, background-free, and angular-independent images of the fiber tip. In addition, in PA, the only part of the fiber being visualized is its tip (not the body). US imaging visualizes the anatomical structure of the background tissue. However, US imaging is unable to visualize the fiber tip in angled orientations because it suffers from angular dependency. The supplementary movies indicate

i) 3D volumetric image of the fiber tip in straight orientation,

ii) 3D volumetric image of the fiber tip in angled orientations using

a. Combined US and PA imaging (US shows the background and PA indicates the location of the fiber tip).

b. US image is not able to locate the fiber.

B. Ablation fiber carrying the combined continuous wave (CW) and pulsed beams

The puled laser beam at λ=532 nm and CW laser beam at λ=808 nm was coupled using a short pass dichroic mirror with cut-off wavelength of 650 nm into a single ablation fiber for simultaneous ablation and PA imaging. A dichroic mirror transmits pulsed beam (532 nm) and reflects CW beam (808 nm) and thus allowing for combining the beams into a single fiber. The supplementary movie shows the combined beam exiting a single ablation fiber.
